# Immune system modulation in aging: Molecular mechanisms and therapeutic targets

**DOI:** 10.3389/fimmu.2022.1059173

**Published:** 2022-12-15

**Authors:** Bulmaro Cisneros, Ian García-Aguirre, Juan Unzueta, Isabel Arrieta-Cruz, Oscar González-Morales, Juan M. Domínguez-Larrieta, Aura Tamez-González, Gerardo Leyva-Gómez, Jonathan J. Magaña

**Affiliations:** ^1^ Departamento de Genética y Biología Molecular, Centro de Investigación y de Estudios Avanzados, Instituto Politécnico Nacional, Ciudad de México, Mexico; ^2^ Departamento de Bioingeniería, Escuela de Ingeniería y Ciencias, Tecnologico de Monterrey, Ciudad de México, Mexico; ^3^ Departamento de Investigación Básica, División de Investigación, Instituto Nacional de Geriatría, Secretaría de Salud, Ciudad de México, Mexico; ^4^ Departamento de Bioingeniería, Escuela de Ingeniería y Ciencias, Tecnologico de Monterrey, Jalisco, Mexico; ^5^ Departamento de Bioingeniería, Escuela de Ingeniería y Ciencias, Tecnologico de Monterrey, Nuevo León, Mexico; ^6^ Departamento de Farmacia, Facultad de Química, Universidad Nacional Autónoma de México, Ciudad de México, Mexico; ^7^ Laboratorio de Medicina Genómica, Departamento de Genética, Instituto Nacional de Rehabilitación “Luis Guillermo Ibarra Ibarra”, Secretaría de Salud, Ciudad de México, Mexico

**Keywords:** immunosenescence, inflammaging, chronic infections, aging, immune system

## Abstract

The function of the immune system declines during aging, compromising its response against pathogens, a phenomenon termed as “immunosenescence.” Alterations of the immune system undergone by aged individuals include thymic involution, defective memory T cells, impaired activation of naïve T cells, and weak memory response. Age-linked alterations of the innate immunity comprise perturbed chemotactic, phagocytic, and natural killing functions, as well as impaired antigen presentation. Overall, these alterations result in chronic low-grade inflammation (inflammaging) that negatively impacts health of elderly people. In this review, we address the most relevant molecules and mechanisms that regulate the relationship between immunosenescence and inflammaging and provide an updated description of the therapeutic strategies aimed to improve immunity in aged individuals.

## 1 Introduction

The worldwide population is suffering an accelerated growth of old people, bringing formidable healthcare and socioeconomic challenges. From a biological perspective, aging is a complex and multisystemic process that adversely impacts on the organism function, with the nervous, endocrine, hematopoietic, and immune systems being the most affected by this process. Specifically, aging elicits a decline in the immune system, affecting both the innate and adaptive immunity responses (immunosenescence), which result in increased vulnerability to toxins and pathogens and the establishment of a chronic inflammation state (inflammaging). An in-depth study of the immune system has regained relevance due to the public health emergency caused by the coronavirus disease 2019 (COVID-19) pandemic, which preferentially affects older individuals. In this scenario, the present review is focused on the mechanism underlying both immunosenescence and inflammaging, providing also a description of current therapeutic strategies aimed to ameliorate the impact of aging/senescence on immunity.

## 2 Immunosenescence

**Figure 1:**
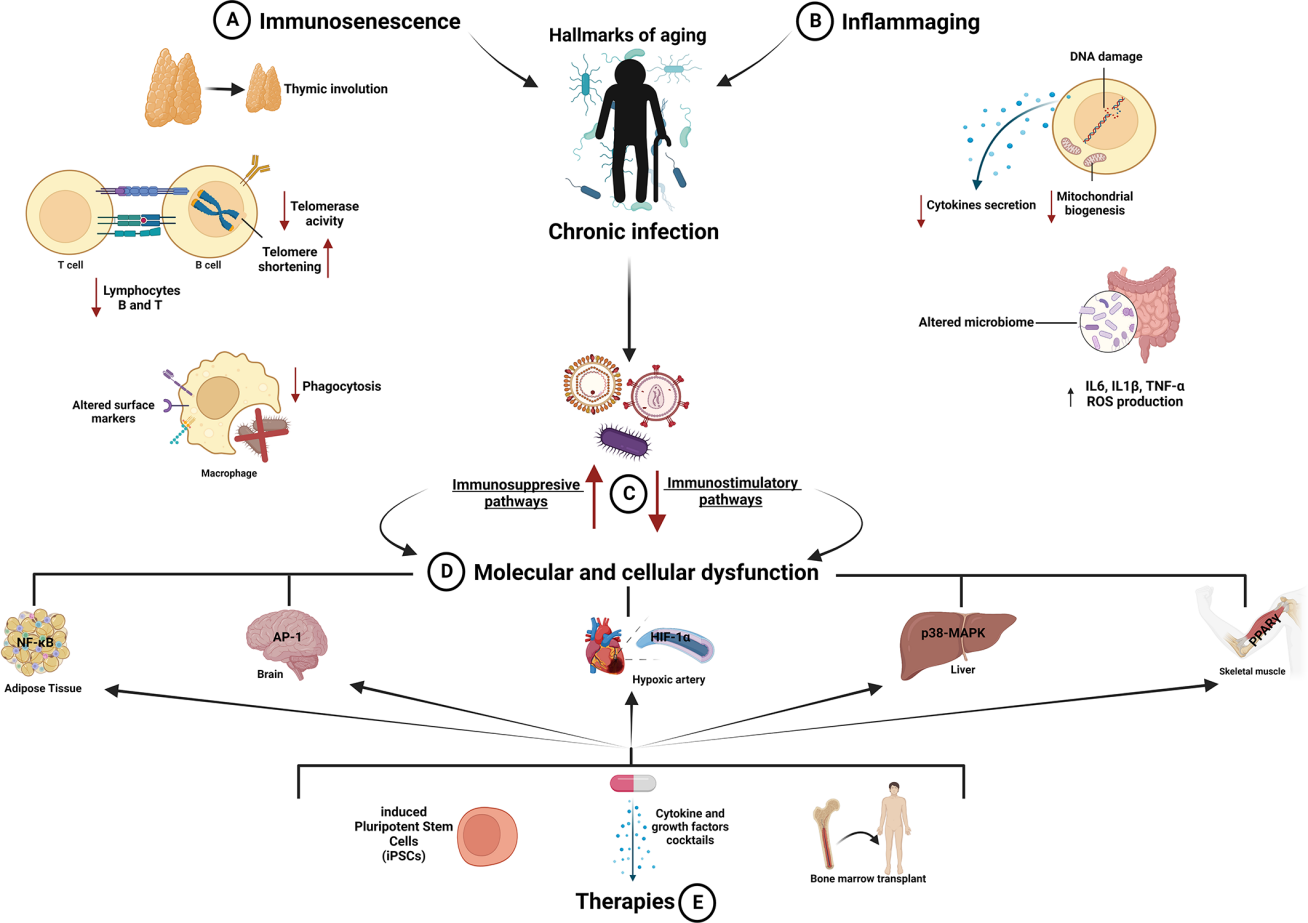
Aging of the immune system: mechanisms and therapeutic strategies. **(A, B)** The immune system declines during aging, and its exposure to pathogens induces overstimulation and overreaction of immune cells (macrophages; lymphocytes B and T; natural killer and dendritic cells), releasing chemical mediators that affect their function and driving them toward immunosenescence and inflammaging. These processes accelerate the onset of age-related diseases reducing health span. **(C)** Aging alters immunity provoking unbalance between immunostimulatory and immunosuppressive mechanisms, which in turn impairs relevant functions of the immune system, including thymic involution, altered surface markers and phagocytosis of macrophages, decreased number and activity of B and T lymphocytes, telomerase shortening and DNA damage, reduced cytokine secretion, decreased mitochondrial biogenesis, and elevated ROS level. **(D)** All these changes ultimately cause dysfunction in different tissues and systems such as adipose, hepatic, and skeletal muscle tissues and cardiovascular and nervous systems. **(E)** Therapeutic strategies aimed to rejuvenate the immune system and decrease the risk of infectious diseases in elderly people are depicted. AP-1, activator protein 1; HIF-1α, hypoxia-inducible factor 1; IL1β, interleukin-1β; IL6, interleukin-6; NF-κB, nuclear factor kappa B; p38-MAPK, p38-mitogen activated protein kinase; PPAR-γ, peroxisome proliferator-activated receptor gamma; TNF-α, tumor necrosis factor-alpha. Created with BioRender.

The term “immunosenescence” comprises several humoral and cellular events that generate age-related dysfunction of the immune system (1). This condition is associated with a higher risk of developing different aged-related pathologies, including infections, cardiovascular and neurodegenerative diseases, autoimmunity, and cancer (2). The main determinants of immunosenescence include genetics, nutrition, sex, race, exercise, and pathogen exposure (2, 3). To better understand immunosenescence, it is necessary to consider the age-driven physiological changes that are related to immunity. Body physical barriers are the first line of defense against pathogens, and in elderly people, the skin becomes thinner and drier, which in turn reduces the amount of fat-soluble defensins. Likewise, the mucosal barrier loses efficiency during aging because the ciliary function is impaired, which consequently facilitates pathogen settlement (4). With respect to the cellular events that underlie immunosenescence, thymic involution, decreased number of T and B lymphocyte cells, impaired telomerase activity, increase of inflammatory mediators, and a weak immune response to vaccination have been consistently observed in aging (5–9). It is worthy to mention that these deficiencies are worsened by exposure to pathogens (2, 3). Furthermore, alterations in the innate immune cells (neutrophils, macrophages, natural killers, dendritic, and mast cells) have been found in aged individuals (**Figure 1A**). Neutrophils display diminished killing capacity, even though their production in the bone marrow remains unchanged and their number in blood is even slightly higher in aging (10). Similarly, the function of natural killer cells is disturbed in aging, although their turnover in the bone marrow diminishes and their baseline number increases (11). Macrophages and dendritic cells show similar phagocytic activity between young and old people; however, both the total number of these cells in peripheral blood and their ability to present antigens and stimulate T cells are defective in elderly people (12, 13). In addition, macrophages show increased inflammatory response (14). Finally, the activation and function of mast cells are altered in aged individuals (15). On the other hand, the repertoire of naïve T and memory B lymphocytes is abundant during childhood, whereas in old age, a decline in B-cell production in the bone marrow (16) and a reduction in the number of T lymphocytes due to thymic involution occurs (13, 17). Altogether, these events result in an overall reduction of immunity in older individuals (18).

## 3 Inflammaging

Inflammaging is defined as a systemic proinflammatory state caused by an imbalance between pro- and anti-inflammatory mechanisms, which provokes in turn increased cytokine production (19). This imbalance elicits a prolonged state of low-grade inflammation (19) characterized by augmented levels of pro-inflammatory mediators, including IL-1β, IL-6, TNF-α, IL-8, and CRP (1) (**Figure 1B**). This phenomenon is a hallmark of aging and is even considered a biomarker of accelerated aging (20). Inflammaging is modulated by multitude interrelated processes; at the physiological level, some relevant factors that can promote inflammaging include physical inactivity, obesity, psychological stress, early life adversity, exposure to xenobiotics, and chronic infections (1, 21). Inflammaging is also considered as a risk factor for several pathologies, including cardiovascular, kidney, and neurodegenerative diseases, type 2 diabetes mellitus, cancer, depression, sarcopenia frailty and infectious diseases (20, 22). Furthermore, several studies correlate inflammaging with the susceptibility of older people to develop COVID-19 with severe complications (1, 23), due to a hyperreactive response to the infectious agent through a massive release of chemical mediators (20, 24, 25). Inflammaging has been recently regarded as an adaptive process that can lead either to healthy aging or to a pathological state, depending on genetic and environmental conditions and lifestyle factors (1, 19, 22). This idea has been reinforced by studies on centenarian populations, in which high levels of inflammatory biomarkers were found to favor longevity *via* their interaction with anti-inflammatory molecules (26, 27). Inflammaging is a dynamic and complex process driven by several age-related molecular mechanisms, rather than having an exclusive connection with the immune system (22). For instance, oxidative stress induces age-related transcriptional changes in genes encoding key components of inflammatory pathways (19). Specifically, the pro-inflammatory secretome of senescent cells can exert paracrine effects on nearby tissues extending the inflammatory state at the organismal level (19). Finally, dysregulation of the microbiome is another important contributor to inflammaging (21). It is believed that amelioration of age-related dysbiosis by probiotic clinical intervention might in turn alleviate inflammaging (14).

## 4 Effect of chronic infections on the development of inflammaging and immunosenescence

Chronic infections are a major health problem that affects millions of people worldwide. The innate immune system of aged people loses the ability to respond to viral infections; it initiates a local inflammatory response but fails to eliminate the virally infected cells (28, 29). Chronic infections trigger persistent adaptive immune responses that generate a pro-inflammatory environment. As this state persists, different alterations emerge, such as downregulation of immune responses, which further aggravates the inflammatory response (28, 29) (**Figure 1C**). This unresponsive immune system, also called immunosenescence, exacerbates the inflammatory response, due to accumulation of inefficient adaptive immune cells, which ultimately causes a physiological decline (30).

## 5 Mechanisms controlling inflammaging and immunosenescence

Changes in the immune system that occur during aging have just started to be understood. The intricate interplay between inflammaging and immunosenescence requires to be deciphered, in order to develop therapeutic interventions aimed to improve/rejuvenate the immune system. In this section, the most relevant proteins/pathways and immune system cells that modulate the host immune response during aging are described.

### 5.1 NF-kB

Nuclear factor kappa B (NF-κB) is a main protagonist of the inflammatory and immune responses. NF-κB responds to various stimuli such as the T- and B-cell receptors (TCR and BCR, respectively) (31). During chronic infections, NF-κB orchestrates different T-cell responses; it induces maturation of T cells in the thymus and modulates differentiation and activation of regulatory T cells (Tregs). These activities are required to induce or suppress the immune response, lessening inflammation (32, 33). Furthermore, NF-κB delays immunosenescence by upregulating telomerase production in T cells during chronic infections, thereby promoting an opportune clonal expansion (34). On the other hand, NF-κB elicits production of IL-6 and TNF-α in macrophages, which contributes to immune clearance, but in the long-term could accelerate inflammaging, provoking cell and tissue damage (**Figure 1D**). Overall, these studies place NF-κB as a neuralgic center where pro-inflammatory and anti-inflammatory signals converge (35–37).

### 5.2 HIF-1α

Hypoxia is a pivotal modulator of immunity. It regulates immune cell proliferation and the response to pathogens through epigenetic regulation, which is largely controlled by transcription factor hypoxia-inducible factor 1 (HIF-1α) (38). When a chronic infection is established, a large amount of reactive oxygen species (ROS), chemokines and cytokines, such as IL-1β, are produced, which in turn increases inflammation and parallelly activates NF-κB-mediated HIF-1α synthesis (39, 40). HIF-1α is a key transcription factor for modulating the inflammation response, because it promotes the expression of proinflammatory cytokines and chemokines (39, 40). Consistent with this role is that HIF-1α-deficient mice were found to be resistant to develop inflammatory diseases; however, when they were subjected to chronic infection developed inflammatory responses and died early, compared with control animals (41–43). Overall, these studies state that hypoxia-mediated HIF-1α activation promotes a chronic low-grade inflammatory state (inflammaging), which in turn can lead to immunosenescence (40). The crucial function of HIF-1α in these phenomena makes it an ideal therapeutic target to modulate immune responses during aging.

### 5.3 Lymphocytes B

Lymphocytes B are the humoral immune response cells responsible for antibody production. These cells provide discrimination between self and non-self-antigens and the memory to evoke previous contact with specific pathogens, which result in a bulkier response in subsequent host–pathogen interactions (44). During persistent viral infections, accumulation of atypical deficient B cells occurs, which are unable to differentiate into antibody productive cells and have also reduced the ability to trigger production of cytokines, antibodies, and the B-cell receptor (45, 46). On the contrary, the response of B cells in the germinal centers is robust and efficient as the infection progress, which contrasts to the persistent T-cell response that leads to their exhaustion (47). The continued immune response generates in turn an exacerbated pro-inflammatory environment, with high production of autoantibodies by B cells. The formation of this inflammation-feedback loop greatly contributes to the establishment of immunosenescence (16, 48). The above-described alterations undergone by B cells that result in a generalized reduction of the overall immunity are faithfully recreated during aging, affecting the protection of the elderly against pathogens (49) (**Figure 1D**).

### 5.4 ROS

Oxidative stress emerges as a consequence of the loss of the redox (reduction/oxidation) balance. Then, high levels of ROS cause oxidation of lipids, proteins, and DNA and innate immune responses (Toll-like receptor signaling and NLRP-3 inflammasome) (50). An enhanced expression of cytokines and chemokines (IL-1, IL-6, TNF-α, and IL-18) provokes further augmentation of ROS levels, creating a positive feedback loop of ROS production (50) (**Figure 1B**). Using mouse models of aging, the connection between ROS and immunosenescence has been evidenced. It has been observed that both leukocytes and macrophages from premature aging mice (PAM) lose the balance between oxidant compounds and the antioxidant defense (51). Conversely, long-lived mice maintain the redox equilibrium in macrophages (52). During aging, macrophages produce a high amount of oxidant compounds and lipofuscin to enter consequently into immunosenescence (52). On the other hand, viral infections (HIV or herpes virus) also cause the immune cells to generate oxidant compounds, and when a chronic infection is established, the persistence of oxidative stress leads to chronic inflammation and later to premature immunosenescence (53), *via* NF-κB activation and induction of TNFα, IL6, and IL1 expression (54–58). Thus, elevated levels of ROS are mechanistically linked to immunosenescence and inflammaging.

### 5.5 P38-MAPK

The p38-mitogen-activated protein kinase (p38-MAPK) pathway regulates the balance between inflammatory and anti-inflammatory responses, preventing chronic inflammation and the further establishment of immunosenescence. A connection between p38-MAPK activation, inflammaging, and immunosenescence has been demonstrated using a human model of acute inflammation (59). This study showed that the onset of inflammation progress in a similar manner between young and old subjects; however, the conclusion of the process was clearly disturbed in elderly people, due to a decrease of T-cell immunoglobulin mucin receptor-4 (TIM4), a macrophage receptor that enables the engulfment of apoptotic bodies (efferocytosis). This alteration was found to be mechanistically associated with increased p38-MAPK activity in the macrophages of aged subjects, as TIM4 expression and the resolution of inflammation were rescued through oral administration of p38 inhibitors (59). Consistent with a crucial role for p38-MAPK in inflammaging, the sestrin-dependent activation of p38-MAPK induced a pro-aging phenotype in lymphocytes (60) (**Figure 1D**). Furthermore, chronic inflammation and premature immunosenescence phenotypes, induced by bacterial infections, have been found to be associated with a persistent activation of p38-MAPK (61).

### 5.6 Lymphocytes T

As aging progresses, T lymphocytes undergo functional changes that impact their function. It has been reported that the number of T lymphocytes decreases during aging (62); furthermore, they exhibit low proliferation due to replicative senescence induced by telomere shortening. Consistently, aged individuals exhibit an elevated number of T lymphocytes positive for the senescence-associated beta-galactosidase activity (63). Furthermore, the presence of immunosenescent T cells has been related to chronic inflammation during aging (64). Both accumulation of exhausted non-functional T cells and the presence of chronic infections during aging result in an hyperinflammatory state (65) (**Figure 1A**). Remarkably, the evolution of T lymphocytes, from their development to exhaustion, is driven by two key transcriptional factors, namely, transcription factor 7 (TCF7) and thymocyte selection-associated high-mobility group box (TOX) (66). TCF7 belongs to a DNA-binding protein family termed “HMG box”; it is highly expressed in thymocytes and peripheral naïve T cells and is involved in the development and differentiation of T-lineage cells (67). TCF7 exerts its function by assembling with β-catenin into an active transcription complex, which results in WNT/β-catenin signaling pathway activation and the expression of genes implicated in embryonic development and self-renewal of stem cells at the adult age (68). During chronic viral infections, TCF1 is present in T cells with an exhausted phenotype; interestingly, chronically stimulated T cells, which are positive for TCF7, have the ability to either survive for a long time, self-renew, or proliferate (69). As for TOX, it has been associated with CD8^+^ T-cell exhaustion as well (70). TOX is predominantly expressed in hematopoietic and immune tissues, specifically in CD4+ T and natural killer cells, and its expression is activated by the chronic stimulation of CD8^+^ T cells (70). TOX activity in turn promotes CD8^+^ T-cell exhaustion *via* chromatin remodeling and upregulation of T-cell inhibitory receptors, including protein disulfide isomerase (PDI) (71).

## 6 Trends in therapeutic modulation of inflammaging during immunosenescence

During aging, the immune system declines due to dysregulation and overactivation of its innate and adaptive responses, leading to the onset of inflammation-related chronic diseases highly observed in elderly people (72). For that reason, several pharmacological and cellular/genetic strategies have been developed to slow down or reverse the deleterious effects of immunosenescence on health (73): (a) Induced pluripotent stem cells (iPSC) have been employed to generate hematopoietic cells and/or various specific immune cells; (b) administration of cytokine and growth factor cocktails boosted macrophage function; (c) bone marrow transplantation is a widely used therapy for thymus regeneration (74) (**Figure 1E**); (d) the use of Cdc42 and BATF inhibitors or antioxidants enhances the number and function of lymphoid*-*biased hematopoietic stem cells (75, 76); (e) inhibition of dual specific phosphatases 4 boosts memory CD4+ T-cell function (77, 78); (f) administration of fibroblast growth factor 7 (FGF7) stimulates naive T-cell production and promotes the removal of dysfunctional cells, thereby restoring thymus function (79, 80); and (g) administration of rapamycin improves CD8+ T-cell function (81, 82) (**Figure 1**). Finally, a relevant non-pharmacological strategy that has been proven to enhance immunity is caloric restriction; it delays the accumulation of senescent T cells and stimulates thymopoiesis through the activation of IGF-1 and/or PPAR pathways (83, 84). On the other hand, recent studies have unveiled the relevance of functional foods to ameliorate oxidative stress and inflammation and to improve the metabolism of lipids associated with metabolic diseases, *via* Nrf2 and/or NF-*κ*B signaling pathways (85, 86).

Some of the molecules/pathways that modulate immunosenescence have therapeutic potential. Owing to the crucial role of the activator protein 1 (AP-1) signaling pathway in macrophage-mediated inflammation, targeting of AP-1 has been approached to attenuate inflammation. Transfection of lentiviral siRNA against AP-1 in mice fed with high-fat diet resulted in the alleviation of systemic and hepatic inflammation (87). Interestingly, the use of rosiglitazone, a PPARγ agonist, was found to exert a positive effect on animals with sepsis, decreasing cell death and cardiac inflammation; furthermore, increased fatty acid oxidation and improved insulin resistance were also observed in human skeletal muscle (88). Since aging is a very complex process that involves different biological processes, therapies aimed to modulate inflammaging have to be focused on the synergic effect of more than one compound, to regulate simultaneously different pathways. For instance, a combinatory treatment using three different compounds, rapamycin, acarbose, and 17α-estradiol, converge on the regulation of both ERK1/2 and p38-MAPK pathways (89).

## 7 Conclusions

Inflammation is a key factor for the onset and progression of almost all chronic diseases affecting aged individuals, with immunosenescence and inflammaging being two relevant phenomena that modulate the immune system during aging. Therefore, identification and characterization of the molecular and cellular mechanisms underlying the immune system dysfunction will surely help to develop effective therapeutic strategies to prevent the negative outcomes of infectious diseases on aged individuals. Recent scientific evidence indicates that different immune system cells, including hematopoietic stem cells, T cells, B cells, NK cells, thymocytes, macrophages, microglia, granulocytes, and dendritic cells, are suitable targets for cellular and genetic therapies. An effective therapy must combine in a balanced manner immunostimulatory and immunosuppressive strategies, toward a reasonable immune rejuvenation. Given the intricate network of the molecular events involved in the regulation of inflammation/immunosenescence, the therapeutic approaches described herein are focused on the improvement of the immune system in aged individuals rather than longevity.

## Author contributions

BC, GL-G, and JM conceptualized the work. BC, IG-A, JU, IA-C, OG-M, JD-L, AT-G, GL-G, and JM wrote the draft manuscript. JU, IG-A, IA-C, OG-M, JD-L and AT-G designed the figure. BC, IA-C, GL-G and JM supervised and edited the final version of manuscript. BC, GL-G and JM obtained funding. All authors contributed to the final version of manuscript and approved the submitted version.
